# Relationship between Perceived Pain Interference and Poor Psychological Wellbeing among United States Adults

**DOI:** 10.3390/bs13030240

**Published:** 2023-03-09

**Authors:** David R. Axon, Ann Kim

**Affiliations:** 1 Department of Pharmacy Practice & Science, R. Ken Coit College of Pharmacy, The University of Arizona, Tucson, AZ 85721, USA; 2 Center for Health Outcomes and PharmacoEconomic Research (HOPE Center), R. Ken Coit College of Pharmacy, The University of Arizona, Tucson, AZ 85721, USA

**Keywords:** psychological wellbeing, pain severity, pain, US adults, survey research

## Abstract

The goal of this observational project was to investigate the association among perceived pain interference and poor psychological wellbeing in United States adults. Adults over 18 years of age in the 2019 Medical Expenditure Panel Survey were eligible for inclusion if they were alive for the calendar year and had data available for their pain status. Hierarchical logistical regression examined statistically significant associations among perceived pain interference and poor psychological wellbeing. Results showed that greater levels of perceived pain interference were significantly related with larger odds of reporting poor psychological wellbeing. Additionally, several other variables were related with larger or lower odds of reporting poor psychological wellbeing. These findings provide insight into the effect of perceived pain interference and other variables with poor psychological wellbeing, which may help recuperate the psychological wellbeing of US adults with pain.

## 1. Introduction

Psychological wellbeing is a core component of mental health that can affect how an individual thinks, feels, and acts [1]. Psychological wellbeing is increasingly recognized as a critical component of one’s health that, if neglected, can lead to varying degrees of impairment in function, psychosocial disabilities, and clinical outcomes. Issues with psychological wellbeing are prevalent in the United States (US) with the added stressors from the coronavirus disease 2019 (COVID-19), and approximately 50 million US individuals reporting poor psychological wellbeing in 2022 [2,3,4]. Globally, poor psychological wellbeing costs an estimated $1 trillion in lost productivity annually with depression being a primary reason for disability according to the World Health Organization (WHO) [1]. Nevertheless, psychological wellbeing issues are commonly overlooked as there are still over 27 million adults in the US not receiving the necessary treatment [2].

Among numerous factors that affect psychological wellbeing, it has been found that pain often occurs alongside common psychological wellbeing issues [5]. Pain is often described as “an unpleasant sensory and emotional experience associated with, or resembling that associated with, actual or potential tissue damage” [6] and can be affected by various factors that affect social and psychological well-being [7]. Pain is a complex condition that can restrict usual activities, affect perceived health, and reduce quality of life [8]. Pain is among the top reasons for seeking medical attention, with roughly 50 million American adults having chronic pain in 2019 [8]. Although various pain management strategies such as analgesics, anticonvulsants/antidepressants, and nonpharmacological methods are available, many individuals still find their pain management unsatisfactory. A deeper look into factors associated with pain may further studies on multidomain pain management strategies and how they relate to outcomes [9,10].

The relationship between pain and psychological wellbeing indicates individuals with pain have a larger risk for poorer psychological wellbeing due to overlapping neural mechanisms [11]. Studies have also reported that addressing poor psychological wellbeing may be a significant approach for pain management due to its complex psychosocial aspects [12]. Although there is literature on the relationship and healthcare costs of pain and psychological wellbeing in older US adults [13,14], there are limited studies on the relationship among perceived pain interference and psychological wellbeing in US adults regardless of age. The primary objective of this project was to assess the association among perceived pain interference and poor psychological wellbeing in US adults. A secondary objective was to identify any other variables (beyond perceived pain interference) that are associated with poor psychological wellbeing among US adults.

## 2. Methods

### 2.1. Study Design, Data Source, and Study Participants

This observational retrospective database study used survey data from the Medical Expenditure Panel Survey (MEPS) Household Component. Conducted by the Agency for Healthcare Research and Quality, MEPS collects data using large-scale surveys administered to US households. These surveys are administered five times over two full calendar years. Information from these surveys include demographics, economic variables, health conditions, health status, healthcare coverage, healthcare access, and healthcare satisfaction. MEPS staff collate gathered information and create data files that can be used for data analysis. Adults alive and aged 18 years of age or older in the MEPS 2019 full-year consolidated data were included in the project if they had data available for their pain status. MEPS respondents voluntarily participated by providing oral informed consent [15]. The Institutional Review Board at the University of Arizona approved this project (protocol #00001768, 26 August 2022). This research report was prepared following the strengthening the reporting of observational studies in epidemiology (STROBE) guidelines [16].

### 2.2. Independent Variable

The independent variable was perceived pain interference determined by the survey question “During the past 4 weeks, how much did pain interfere with your normal work (including both work outside the home and housework)?”. Available responses incorporated ‘not at all’, ‘a little bit’, ‘moderately’, ‘quite a bit’, and ‘extremely’ [15].

### 2.3. Control Variables

Possible confounders were grouped by applying Andersen’s Behavioral Model of Health Services Use [17] and controlled for using adjusted analyses. Predisposing confounders included age (≥65, 40–64, 18–39); sex (male, female), race (white, not white), and ethnicity (Hispanic, not Hispanic). Enabling confounders included marriage status (married, not married), income status (poor/near poor/low, moderate/high), education status (up to and including high school, more than high school), employment status (employed, not employed), and health insurance status (private, public, no insurance). Need confounders included instrumental activity of daily living (IADL) limitation (yes, no), activity of daily living (ADL) limitation (yes, no), number of chronic diseases (≥2, <2), overall health status (good, poor); regular exercise (yes, no), and smoking status (yes, no) [15].

### 2.4. Dependent Variable

The dependent variable was psychological wellbeing determined by the survey item “In general, would you say that your mental health is excellent, very good, good, fair, or poor?”. Answers of ‘fair’ or ‘poor’ were coded as poor psychological wellbeing and ‘excellent’, ‘very good’, or ‘good’ were coded as good psychological wellbeing [15].

### 2.5. Data Analysis

Differences between individuals with poor psychological wellbeing and good psychological wellbeing were identified using chi-squared tests. Hierarchical logistic regression analysis was used to explore statistically significant associations among perceived pain interference and poor psychological wellbeing. Good psychological wellbeing was the referent group. The first model was the unadjusted model that included perceived pain interference (independent variable). The second model included perceived pain interference and adjusted for predisposing confounders. The third model included perceived pain interference, predisposing, and enabling confounders. Finally, the fourth model included perceived pain interference, predisposing, enabling, and need confounders. The a priori alpha level was 0.05. Analyses utilized SAS PROC SURVEY commands (v9.4, SAS Institute Inc., Cary, NC, USA). The appropriate weighting variable provided in the MEPS dataset was employed to provide nationally representative approximations of the US population. Cluster and strata variables maintained the MEPS data structure. Taylor series linearization calculated estimates in variance.

## 3. Results

**Figure 1:**
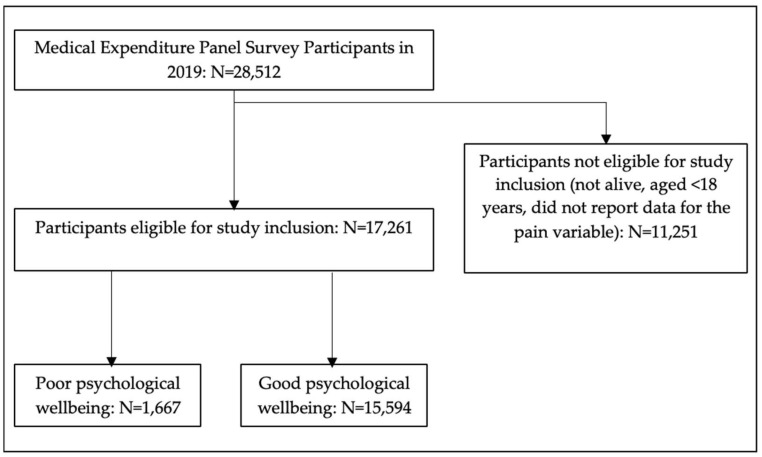
Participant eligibility chart.

Of the 28,512 individuals included in the 2019 MEPS data set, 17,261 were included in this project. Of these, 1667 reported having poor psychological wellbeing and 15,594 reported having good psychological wellbeing. This represented an estimated weighted population of 242,169,897 US adults. Of these, an estimated 20,327,445 reported having poor psychological wellbeing and an estimated 221,842,452 reported having good psychological wellbeing (Figure 1).

In terms of perceived pain interference, among the 17,261 individuals included in the study, 468 recorded extreme pain interference, 1221 recorded quite a bit of pain interference, 1396 recorded moderate pain interference, 3860 recorded little pain interference, and 10,316 recorded no pain interference. This represented an estimated weighted population of 4,950,538 individuals who recorded extreme pain interference, an estimated 14,019,475 individuals who recorded quite a bit of pain interference, an estimated 17,526,400 individuals who recorded moderate pain interference, an estimated 51,964,511 individuals who recorded little pain interference, and an estimated 153,708,972 individuals who recorded no pain interference.

Table 1 shows the characteristics of US adults stratified by poor vs. good psychological wellbeing. The most common age group was 40–64-year-olds. In general, the study sample had an even split among sex, marriage status, and regular exercise status. Most adults in this study were white, not Hispanic, had moderate/high income, had more than high school education, were employed, had private health insurance, had no IADL or ADL limitations, had less than two chronic conditions, had poor overall health, and did not smoke. There was no difference between groups for all variables except race (*p* = 0.6259) and ethnicity (*p* = 0.8514).

Table 2 shows the association of perceived pain interference with poor (vs. good) psychological wellbeing among US adults. In general, our results showed that those who reported having greater levels of perceived pain interference had larger odds of reporting poor vs. good psychological wellbeing. In general, the magnitude of the effect sizes decreased as additional confounders were controlled for in adjusted models. Specifically, in the fully adjusted model (i.e., model 4) those who had quite a bit of pain interference had approximately 2.3 times the odds of reporting poor vs. good psychological wellbeing, while those who had extreme pain interference had two times the odds of reporting poor vs. good psychological wellbeing compared to those who had no pain interference. Those who had moderate pain interference had approximately 1.8 times the odds of reporting poor vs. good psychological wellbeing while those who had little pain interference had 1.6 times the odds of reporting poor vs. good psychological wellbeing compared to those who had no pain interference.

The following variables also showed an association with larger odds of reporting poor vs. good psychological wellbeing in the fully adjusted model (i.e., model 4): race (white vs. not white), income status (poor/low vs. moderate/high), health insurance status (public vs. no insurance), IADL limitation (yes vs. no), ADL limitation (yes vs. no), number of chronic diseases (≥2 vs. <2), and smoking status (yes vs. no). The following variables showed an association with lower odds of reporting poor vs. good psychological wellbeing: age (≥65 and 40–64 vs. 18–39), marriage status (married vs. not married), employment status (employed vs. not employed), overall health status (good vs. poor), and regular exercise (yes vs. no).

## 4. Discussion

There were two key findings from this observational study. The first key finding was that perceived pain interference was significantly associated with psychological wellbeing wherein greater levels of perceived pain interference had larger odds of reporting poor psychological wellbeing. Studies in other countries have indicated similar findings. For example, one nationwide study from Iceland found that people with chronic musculoskeletal pain had a larger risk of poor psychological wellbeing [18]. Another study in China found that university students with chronic pain had more anxiety and depression [19]. Because pain is associated with various factors, there are some explanations why this may be the case. One 2020 study exploring executive functions and pain found that regardless of depression or chronic pain, pain severity was correlated with mental flexibility [20]. Although many studies have looked at specific types of pain and psychological wellbeing, this study reports that any pain correlates with deficits in mental flexibility which may affect the psychological wellbeing of an individual. Meanwhile, a report using 2017 MEPS data looking at psychological wellbeing in older US adults with pain found that overall health status to be the greatest predictor of psychological wellbeing [13]. Physical health can have direct and indirect effects on functioning which impact psychological wellbeing such as restrictions to exercise and quality of life. Therefore, the association found between perceived pain interference and psychological wellbeing could also be in that pain is a function of overall health and therefore exacerbates psychological wellbeing decline. Regardless, this study’s finding supports the existing literature exploring a need for multidisciplinary approaches to pain and psychological wellbeing such as psychiatry in the context of pain management [12].

The second key finding was that several other variables were related to psychological wellbeing. Variables that showed an association with larger odds of poor psychological wellbeing included race, income status, health insurance status, IADL and ADL limitation, number of chronic diseases, and smoking status. Firstly, there are limited reports on the direct relationship between race and psychological wellbeing although there is data that suggests that minority students have lower odds of reporting poor psychological wellbeing relative to Whites [21]. Such findings could be explained by the disparity in access to healthcare for minorities compared to Whites due to socioeconomic factors and insurance [22]. Another factor could be that barriers exist for culturally different groups where there is organizational bias in psychological wellbeing that could affect awareness and psychological wellbeing [23]. The next finding was that lower income was related to poor psychological wellbeing. In support of this finding, one study used data from the Panel Study of Income Dynamics and found that more income was related to reduced psychological distress [24]. Other literature has looked more broadly at income inequality and found its association with poorer psychological wellbeing [25]. This is relevant to our finding on health insurance status as lower income is associated with being uninsured [26]. One study exploring this topic found that private health insurance was related to a lower risk of developing depression in adults aged 65 and older [27]. Our study had no significant association for private health insurance, which is likely explained by the difference in the sample’s age range. Furthermore, our study found a relationship between public health insurance and poorer psychological wellbeing compared to not being insured. This is interesting given that previous work found expanding public insurance increased availability of healthcare for psychological wellbeing [28]. There are limited reports on the association between public health insurance and psychological wellbeing, thus more research should be done to investigate the outcomes of psychological wellbeing under public insurance expansions. The next finding, that IADL and ADL limitations were associated with poor psychological wellbeing, confirms previous literature [29,30]. In addition, having two or more chronic diseases is associated with having an IADL limitation [30]. Our study found that having multiple chronic conditions was associated with greater odds of poor psychological wellbeing, which is supported by previous data that found the presence of psychological wellbeing disorders increased as number of physical morbidities increased [31]. This highlights the need for psychological wellbeing management along with treating physical comorbidities for individuals with IADL and ADL limitations. Further, our finding that smoking was associated with poor psychological wellbeing is not surprising, given results of studies, such as a meta-analysis, that found stopping smoking was associated with less depression, anxiety, stress, and mood vs. smoking [32].

Several variables were also found to be significantly associated with lower odds of reporting poor psychological wellbeing, including age, marriage status, employment status, overall health status, and regular exercise. Our study found that adults aged 40 and older had lower odds of reporting poor psychological wellbeing compared to adults aged 18–39 years old. This is in line with previous work where depression was found to be less prevalent in older adults than younger adults due to age-related increases in psychological resilience, higher education, and socioeconomic status [33]. Being married was also found to have lower odds of reporting poor psychological wellbeing in our study. Existing literature that found being unmarried was a significant independent predictor of depression later in life confirms our finding [34]. More recent literature found that married individuals had better psychological wellbeing, which suggests marriage-related long term social support and increased economic resources most likely leading to financial satisfaction [35]. Financial satisfaction may also rationalize our finding that employment status was associated with psychological wellbeing. Consequently, other studies support this finding on a global scale, such as one study that found unemployed status among young Korean adults was significantly associated with increased risk of poor psychological wellbeing [36], and another study where unemployment was associated with higher prevalence of poorer psychological wellbeing in Spain [37]. Next, our study found that good overall health status was associated with lower odds of reporting poor psychological wellbeing. Good health status was also found to be the strongest predictor of good psychological wellbeing among older US adults in a further study [13]. This finding aligns with a previous mediation analysis study that found strong indirect effects between mental and physical health [38]. One of these indirect effects was physical activity, which had a positive association with better physical and mental health [38]. This is related to our finding that individuals undertaking regular exercise had lower odds of reporting poor psychological wellbeing. This is further supported by data from another study that found physical activity was positively associated with self-rated health and psychological wellbeing [39]. These key findings from our study provide insight into the significant effects of various variables on psychological wellbeing which may help contribute to the advancement of holistic psychological wellbeing. These findings also suggest that further research is needed to further investigate such variables in greater detail, perhaps using a different dataset.

There are some study limitations to note. This study cannot explain the casual relationship between variables due to the cross-sectional nature of the project. Another limitation was the possibility of bias from editing of data and bias from reporting errors given the self-reported nature of MEPS. In particular, the topic of pain and psychological wellbeing is broad in interpretation leading to difficulties in conveying in scaled answers. In addition, this study made use of secondary data and was therefore limited by the available data. In some cases, alternative variables would have been preferred (e.g., partnership status rather than marital status). Several variables were dichotomized (e.g., ethnicity) where more detailed breakdowns of the data would have been informative, thus limiting the value of the analysis. Therefore, results may vary when compared to other studies. Further work is needed to explore differences in findings within more granular levels of certain variables, for instance smaller age ranges, race/ethnicity, and geographic diversity. Other dataset may be needed to better explore these associations. A prediction model could be developed in future that would include the sensitivity, specificity, and likelihood ratios to better estimate effect sizes. Nonetheless, MEPS provides a large and nationally representative dataset that has good external validity and generalizability. This may be used in conjunction with qualitative studies for better understanding of pain and psychological wellbeing for policymakers and healthcare professionals.

## 5. Conclusions

There were two key findings from this observational study. The first finding was that greater levels of perceived pain interference were significantly associated with larger odds of reporting poor psychological wellbeing. The second finding was that several other variables, including age, race, marriage status, income status, employment status, health insurance status, IADL and ADL limitation, number of chronic diseases, overall health status, regular exercise, and smoking status, were also associated with larger or lower odds of reporting poor psychological wellbeing. These findings provide insight into the effect of perceived pain interference and other variables with poor psychological wellbeing, which may help improve psychological wellbeing for US adults. These findings also advocate for the need to improve the psychological wellbeing of people with pain in the US.

## Figures and Tables

**Table 1:** Characteristics of United States adults stratified by poor vs. good psychological wellbeing.

Variables	Total *n* = 17,261 Weighted Percent (95% CI)	Poor Psychological Wellbeing *n* = 1667 Weighted Percent (95% CI)	Good Psychological Wellbeing *n* = 15,594. Weighted Percent (95% CI)	*p*
Pain interference:				<0.0001
Extreme	2.0(1.8, 2.3)	10.1 (8.5,11.9)	1.3 (1.1, 1.5)	
Quite a bit	5.8 (5.4, 6.2)	18.9 (16.8, 21.0)	4.6 (4.2, 5.0)	
Moderate	7.2 (6.7, 7.7)	13.5 (11.6, 15.3)	6.7 (6.2, 7.1)	
Little	21.5 (20.7, 22.2)	23.8 (21.5, 26.1)	21.2 (20.5, 22.0)	
No pain	63.5 (62.5, 64.4)	33.7 (30.7, 36.6)	66.2 (65.2, 67.2)	
Predisposing:				
Age (years)				0.0012
≥65	21.1 (20.2, 22.1)	25.3 (22.7, 27.9)	20.8 (19.8, 21.7)	
40–64	41.2 (40.2, 42.2)	39.8 (37.0, 42.5)	41.3 (40.3, 42.3)	
18–39	37.6 (36.6, 38.7)	35.0 (31.9, 38.0)	37.9 (36.8, 39.0)	
Sex				<0.0001
Male	48.3 (47.7, 48.9)	42.6 (39.7, 45.4)	48.8 (48.2, 49.4)	
Female	51.7 (51.1, 52.3)	57.4 (54.6, 60.3)	51.2 (50.6, 51.8)	
Race				0.6259
White	77.9 (76.4, 79.4)	78.5 (75.9, 81.1)	77.9 (76.3, 79.4)	
Not white	22.1 (20.6, 23.6)	21.5 (18.9, 24.1)	22.1 (20.6, 23.7)	
Ethnicity				0.8514
Hispanic	16.6 (14.9, 18.3)	16.4 (13.6, 19.1)	16.6 (14.9, 18.3)	
Not Hispanic	83.4 (81.7, 85.1)	83.6 (80.9, 86.4)	83.4 (81.7, 85.1)	
Enabling:				
Marriage status				<0.0001
Married	52.1 (50.9, 53.3)	37.2 (34.0, 40.5)	53.5 (52.3, 54.6)	
Other	47.9 (46.7, 49.1)	62.8 (59.5, 66.0)	46.5 (45.4, 47.7)	
Income status				<0.0001
Poor/low	26.1 (24.9, 27.4)	46.0 (42.3, 49.7)	24.3 (23.1, 25.6)	
Moderate/high	73.9 (72.6, 75.1)	54.0 (50.3, 57.7)	75.7 (74.4, 76.9)	
Education status				<0.0001
Up to and including high school	39.5 (38.0, 41.1)	52.6 (49.4, 55.9)	38.3 (36.8, 39.8)	
More than high school	60.5 (58.9, 62.0)	47.4 (44.1, 50.6)	61.7 (60.2, 63.1)	
Employment status				<0.0001
Employed	68.0 (67.0, 69.0)	46.7 (43.4, 50.0)	70.0 (69.0, 71.0)	
Not employed	32.0 (31.0, 33.0)	53.3 (50.0, 56.6)	30.0 (29.0, 31.0)	
Health insurance				<0.0001
Private	68.8 (67.4, 70.3)	49.2 (45.8, 52.6)	70.6 (69.3, 72.0)	
Public	23.6 (22.5, 24.8)	45.0 (41.7, 48.3)	21.7 (20.6, 22.7)	
Not insured	7.5 (6.7, 8.3)	5.8 (4.3, 7.2)	7.7 (6.8, 8.5)	
Need:				
IADL Limitation				<0.0001
Yes	3.2 (2.9, 3.5)	15.7 (13.6, 17.7)	2.0 (1.8, 2.3)	
No	96.8 (96.5, 97.1)	84.3 (82.3, 86.4)	98.0 (97.7, 98.2)	
ADL Limitation				<0.0001
Yes	1.9 (1.6, 2.1)	10.3 (8.6, 12.0)	1.1 (0.9, 1.3)	
No	98.1 (97.9, 98.4)	89.7 (88.0, 91.4)	98.9 (98.7, 99.1)	
Number of chronic diseases				<0.0001
≥2	42.0 (41.0, 43.1)	63.6 (60.7, 66.5)	40.1 (39.0, 41.1)	
<2	58.0 (56.9, 59.0)	36.4 (33.5, 39.3)	59.9 (58.9, 61.0)	
Overall health				<0.0001
Good	88.1 (87.4, 88.8)	40.8 (37.6, 43.9)	92.4 (91.9, 92.9)	
Poor	11.9 (11.2, 12.6)	59.2 (56.1, 62.4)	7.6 (7.1, 8.1)	
Regular exercise				<0.0001
Yes	51.0 (49.8, 52.2)	31.2 (28.3, 34.0)	52.8 (51.6, 54.0)	
No	49.0 (47.8, 50.2)	68.8 (66.0, 71.7)	47.2 (46.0, 48.4)	
Smoking status				<0.0001
Yes	14.0 (13.2, 14.9)	24.2 (21.5, 27.0)	13.1 (12.3, 13.9)	
No	86.0 (85.1, 86.8)	75.8 (73.0, 78.5)	86.9 (86.1, 87.7)	

This analysis was based on an unweighted sample of 17,261 United States adults age ≥18 years alive during 2019. Differences between groups were assessed using a chi-square test. 95% CI = 95% confidence interval. IADL = instrumental activities of daily living. ADL = activities of daily living.

**Table 2:** Association of perceived pain interference with poor (vs. good) psychological wellbeing in United States adults.

Factor	Model 1 OR (95% CI)	Model 2 OR (95% CI)	Model 3 OR (95% CI)	Model 4 OR (95% CI)
Pain interference:				
Extreme	**15.4 (12.2, 19.5)**	**18.5 (14.7, 23.3)**	**11.3 (8.9, 14.4)**	**2.0(1.4, 2.9)**
Quite a bit	**8.1 (6.7, 9.8)**	**9.7 (7.9, 12.0)**	**7.0 (5.6, 8.6)**	**2.3 (1.8, 2.9)**
Moderate	**4.0 (3.3, 4.8)**	**4.7 (3.9, 5.7)**	**3.8 (3.1, 4.6)**	**1.8 (1.4, 2.3)**
Little	**2.2 (1.9, 2.6)**	**2.4 (2.1, 2.9)**	**2.3 (2.0, 2.7)**	**1.6 (1.3, 1.9)**
No pain	Reference			
Predisposing:				
Age (years)				
≥65		**0.6 (0.5, 0.7)**	**0.5 (0.4, 0.6)**	**0.4 (0.3, 0.5)**
40–64		**0.7 (0.6, 0.8)**	**0.7 (0.6, 0.9)**	**0.5 (0.5, 0.7)**
18–39	Reference			
Sex				
Male		0.9 (0.8, 1.0)	0.9 (0.8, 1.1)	0.9 (0.8, 1.0)
Female	Reference			
Race				
White		1.0 (0.9, 1.2)	**1.2 (1.0, 1.5)**	**1.3 (1.1, 1.6)**
Not white	Reference			
Ethnicity				
Hispanic		**1.2 (1.0, 1.4)**	1.0 (0.8, 1.2)	1.0 (0.8, 1.2)
Not Hispanic	Reference			
Enabling:				
Marriage status				
Married			**0.6 (0.6, 0.7)**	**0.7 (0.6, 0.8)**
Other	Reference			
Income status				
Poor/low			**1.4 (1.2, 1.7)**	**1.3 (1.1, 1.5)**
Moderate/high	Reference			
Education status				
Up to and including high school			**1.2 (1.0, 1.3)**	1.1 (0.9, 1.3)
More than high school	Reference			
Employment status				
Employed			**0.6 (0.5, 0.7)**	**0.8 (0.7, 1.0)**
Not employed	Reference			
Health insurance status				
Private			1.2 (0.9, 1.6)	1.2 (0.9, 1.6)
Public			**1.8 (1.3, 2.4)**	**1.5 (1.1, 2.0)**
not insured	Reference			
Need:				
IADL Limitation				
Yes				**1.8 (1.3, 2.4)**
No	Reference			
ADL Limitation				
Yes				**1.8 (1.3, 2.6)**
No	Reference			
Number of chronic diseases				
≥2				**1.3 (1.0, 1.6)**
<2	Reference			
Overall health				
Good				**0.1 (0.1, 0.1)**
Poor	Reference			
Regular exercise				
Yes				**0.7 (0.6, 0.8)**
No	Reference			
Smoking status				
Yes				**1.3 (1.1, 1.6)**
No	Reference			

The analysis was based on an unweighted sample of 17,261 United States adults age ≥18 years alive during 2019. Model 1 was an unadjusted model that included only perceived pain interference (Wald statistic: *p* < 0.0001; c-statistic: 0.715). Model 2 included perceived pain interference and was adjusted for predisposing confounders (Wald statistic: *p* < 0.0001; c-statistic: 0.730). Model 3 included perceived pain interference and was adjusted for predisposing and enabling confounders (Wald statistic: *p* < 0.0001; c-statistic: 0.770). Model 4 included perceived pain interference and was adjusted for predisposing, enabling, and need confounders (Wald statistic: *p* < 0.0001; c-statistic: 0.856). 95% CI = 95% confidence interval. IADL = instrumental activities of daily living. ADL = activities of daily living. Bold indicates the variable had a significant association.

## Data Availability

Data are available from the corresponding author upon reasonable request.
